# Physiological and Metabolic Effects of 5-Aminolevulinic Acid for Mitigating Salinity Stress in Creeping Bentgrass

**DOI:** 10.1371/journal.pone.0116283

**Published:** 2014-12-31

**Authors:** Zhimin Yang, Zuoliang Chang, Lihong Sun, Jingjin Yu, Bingru Huang

**Affiliations:** 1 College of Agro-Grassland Science, Nanjing Agricultural University, Nanjing, 210095, PR China; 2 Department of Plant Biology and Pathology, Rutgers University, New Brunswick, New Jersey, 08901, United States of America; Zhejiang University, China

## Abstract

The objectives of this study were to determine whether foliar application of a chlorophyll precursor, 5-aminolevulinic acid (ALA), could mitigate salinity stress damages in perennial grass species by regulating photosynthetic activities, ion content, antioxidant metabolism, or metabolite accumulation. A salinity-sensitive perennial grass species, creeping bentgrass (*Agrostis stolonifera*), was irrigated daily with 200 mM NaCl for 28 d, which were foliar sprayed with water or ALA (0.5 mg L^−1^) weekly during the experiment in growth chamber. Foliar application of ALA was effective in mitigating physiological damage resulting from salinity stress, as manifested by increased turf quality, shoot growth rate, leaf relative water content, chlorophyll content, net photosynthetic rate, stomatal conductance and transpiration rate. Foliar application of ALA also alleviated membrane damages, as shown by lower membrane electrolyte leakage and lipid peroxidation, which was associated with increases in the activities of antioxidant enzymes. Leaf content of Na^+^ was reduced and the ratio of K^+^/Na^+^ was increased with ALA application under salinity stress. The positive effects of ALA for salinity tolerance were also associated with the accumulation of organic acids (α-ketoglutaric acid, succinic acid, and malic acid), amino acids (alanine, 5-oxoproline, aspartic acid, and *γ* -aminobutyric acid), and sugars (glucose, fructose, galactose, lyxose, allose, xylose, sucrose, and maltose). ALA-mitigation of physiological damages by salinity could be due to suppression of Na^+^ accumulation and enhanced physiological and metabolic activities related to photosynthesis, respiration, osmotic regulation, and antioxidant defense.

## Introduction

Salinity stress is becoming a major factor limiting plant growth in areas with increasing use of non-potable water sources for irrigation. Excessive salt in the soil leads to a series of physiological and biochemical metabolic disorders in plants, mainly as a result of osmotic effects (dehydration) and nutritional imbalance and Na^+^ toxicity [1]. Physiological and biochemical effects of salinity stress are characterized by internal water deficit, decline in photosynthetic activities and membrane stability, as well as induction of oxidative damages [2], [3]. Understanding metabolic factors that may suppress the adverse effects of salinity stress is critically important for developing salinity-tolerant plant germplasm and improving salinity tolerance in salt-affected areas.

Plant growth regulators (PGR) are known to play roles in regulating plant tolerance to various abiotic stresses. Recent research has found that a key precursor in the biosynthesis of porphyrins in chlorophyll and hemes, 5-aminolevulinic acid (ALA), exhibits PGR properties, promoting plant growth under normal environments and stressful conditions when applied at low concentrations [4], [5]. Exogenous application of ALA at low concentrations increases leaf chlorophyll content, promoting photosynthetic capacity and yield of crops [6], [7]. Improved salinity tolerance with ALA application have been reported mostly in dicot species, such as cotton (*Gossypium hirsutum*) (foliar spray at 10–100 mg·L^−1^) [6], oilseed rape (*Brassica napus*) (foliar spray at 30 mg·L^−1^) [8], [9], potato (*Solanum tuberosum*) (0.3–3 mg·L^−1^ in culture medium) [10], and spinach (*Spinacia oleracea*) (foliar spray at 0.6 and 1.8 mM ALA) [11]. Watanabe et al. [6] reported that among 12 representative PGRs examined, ALA was the most effective in promoting plant tolerance to salinity in cotton. ALA was also found to enhance plant tolerance to drought [12] and heavy metals [13], [14]. The positive effects of ALA on salinity tolerance have been associated with the reduction in Na^+^/K^+^ ratio both in roots and leaves [8], increases in chlorophyll content and photosynthetic rate [8], [15], elevated activities of enzymatic or non-enzymatic antioxidant systems providing significant protection to membranes against harmful reactive oxygen species within tissues [11], or reducing stomatal limitation to gas exchange [7]. Despite the knowledge of the beneficial effects of ALA on stress tolerance, there is lack of comprehensive understanding of physiological and biochemical factors responsive to ALA that may mediate salinity tolerance, such as metabolic changes, in addition to changes in photosynthesis, ion content and antioxidant enzymes. Little was known about effects of ALA on the accumulation of metabolites, such as sugars, amino acids, and organic acids. In addition, the effectiveness of ALA for improving salinity tolerance varies with plant species and dose requirements also differ between plant species, as described above. The effective doses for promoting perennial grass tolerance to salinity have yet to be determined.

Many perennial grasses, such as creeping bentgrass (*Agrostis stolonifera*) commonly used as turfgrass, are sensitive to salinity stress [16], [17]. Several studies found that ALA at low concentration enhanced turfgrass quality, growth and photosynthetic rate of St. Augustine grass (*Stenotaphrum secundatum*), manilagrass (Z*oysia matrella*), and creeping bentgrass under favorable environmental conditions [18], [19]. However, how ALA application may affect perennial grass responses to salinity stress and the effective doses of ALA affecting perennial grass tolerance to salinity stress are not well documented. Such information is important for further understanding mechanisms regulating salinity tolerance and for developing effective chemical products and management strategies to promote plant growth under salinity stress. Therefore, the objectives of this study were to evaluate growth and physiological effects of ALA application under salinity stress in creeping bentgrass, a widely-used, salinity-sensitive turfgrass species, and to examine whether improved stress tolerance was associated with the reduction in Na^+^ accumulation, the mitigation of membrane damages associated with enhancement in the activity of major antioxidant enzymes, and/or changes in metabolite accumulation associated with energy, metabolism, and osmotic regulation.

## Materials and Methods

### Plant materials and growth conditions

Tillers of ‘L-93’ creeping bentgrass were transplanted into polyvinyl chloride pots (20 cm in diameter, 18 cm in height) filled with fine sand on March 1, 2012. Plants were maintained for 50 d in a greenhouse in Nangjing Agricultural University, China, with average daily temperature of 18°C and approximately 11-h photoperiod of natural sunlight. After 50-d plant establishment, plants were moved to a growth chamber (3×2 m) set at 23/18°C (day/night), a 12-h photoperiod with a photosynthetically active radiation (PAR) level of 600 µmol·m^−2^·s^−1^ at the plant canopy level. Plants in the greenhouse or in the growth chamber during the establishment period were irrigated twice per week until water drained from the bottom of the pot to maintain soil water content at the pot capacity level, and were fertilized weekly with 500 ml of half-strength Hoagland's nutrient solution [20].

### Treatments and experimental design

The experiment consisted of two factors (ALA and salinity, each with a non-treated control) with four treatment combinations. In treatment one (designated as non-salinity, untreated), plants were maintained well-watered and fertilized with half-strength Hoagland's nutrient solution. In treatment two (designated as non-salinity+ALA), plants that were maintained well-watered and fertilized with half-strength Hoagland's nutrient solution were foliar sprayed daily with 50 ml ALA at 0.5 mg L for 3 d and then weekly. This application rate of ALA was found to be the most effective dose in promoting salinity tolerance based on turf quality (TQ) in preliminary tests using different concentrations (0.25, 0.5, 1, and 2 mg L^−1^) (data not shown). In treatment three (designated as salinity), plants were gradually exposed to salinity by soil drenching with 500 ml of 50 mM and 100 mM NaCl dissolved in half-strength Hoagland's nutrient solution for 3 d at each concentration, and then with 200 mM as the final concentration to prevent initial salinity shock, and then irrigated daily for 28 d with 500 ml of 200 mM NaCl solution. In treatment four (designated as salinity+ALA), salinity-treated plants were foliar sprayed daily with ALA at 0.5 mg L^−1^ for 3 d prior to the imposition of salinity treatment and then weekly during salinity treatment. The control plants for ALA under non-salinity or salinity conditions were sprayed with water in the same volume as the ALA solution.

Treatments were arranged as a completely randomized design with two factors (ALA and salinity) or four treatments (untreated, ALA-treated, salinity, and salinity+ALA). Each treatment had four replicates (four pots with multiple plants in each pot). The significance of treatment effects and the interactions of ALA and salinity at different treatment days in all parameters measured was tested using the two-way analysis of variance (ANOVA) using the SPSS program (Version 13.0, SPSS Inc., IL, USA). Treatment effects and interactions were significant across different days of treatment based on ANOVA and therefore, differences among treatments at a given day of treatment were separated by Fisher's protected least significance difference test with *P* = 0.05.

### Growth and physiological measurements

Shoot growth was evaluated by visual rating of TQ and the measurement of vertical shoot growth rate (SGR). Visual TQ was rated on a scale of 1 to 9 with 1 being completely desiccated and brown turf canopy and 9 representing healthy plants with dark-green turgid leaves and a dense turf canopy [21]. A rating of 6 was considered the minimal acceptable TQ level. Shoot growth rate was calculated as the difference between the canopy heights within 4 d intervals and divided by 4-d for average daily growth.

Water status of plants was evaluated as leaf relative water content (RWC) weekly during salinity treatment, which was calculated using the formula: 100×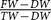 where FW is leaf fresh weight, TW is leaf turgid weight, and DW is leaf dry weight. The FW was determined immediately after leaves were detached from the plant. The TW was determined after leaves were soaked in deionized water for 24 h and blotted dry. The DW was measured following drying of leaves for 72 h in an oven at 80°C.

For the determination of leaf chlorophyll content (Chl), fresh leaves (0.2 g) were detached from plants and then soaked in dimethyl sulfoxide in the dark for 72 h. The absorbance of the chlorophyll extractant was measured at 663 and 645 nm using a spectrophotometer (Ultrospec 2100, Biochrom LTD, UK). The content of Chl was calculated as described in Arnon [22].

Photosynthetic parameters including leaf net photosynthetic rate (Pn), transpiration rate (Tr), and stomatal conductance (g_s_) were measured on 10 full-expanded intact leaves per pot which were enclosed in a leaf chamber with a built-in red and blue light source at PAR of 800 µmol·m^−2^·s^−1^ in the gas exchange analyzer (Li-6400, LICOR, Inc., Lincoln, NE, USA). The chamber CO_2_ concentration was controlled at 390 ppm using an automatic CO_2_ controller in the Li-6400 system.

### Membrane stability, lipid peroxidation, and antioxidant enzyme activity

Leaf membrane stability was evaluated weekly during salinity treatment by measuring electrolyte leakage of leaves [23]. Fresh leaves (0.1–0.2 g) were collected, rinsed, and immersed in 30 ml deionized water and placed on a shaker at 23°C for 24 h. The initial conductivity of the solution (C_initial_) was then measured using a conductivity meter (Orion Star A212, Thermo Scientific Inc., USA). Leaves were then killed by autoclaving at 120°C for 20 min and placed back on the shaker for 12 h. The final conductivity of killed tissues (C_max_) was then measured and electrolyte leakage (EL) calculated as the percentage of C_initial_ over C_max_ [23].

Antioxidant enzyme activity was determined at 28 d of salinity stress using the method described by Zhang and Kirkham [24]. Fresh leaf tissues (0.40 g) were ground to a fine powder using a mortar and pestle and extracted with 5 ml extraction buffer (50 mM potassium phosphate, 1 mM ethylenediaminetetraacetic acid, 1% polyvinylpyrrolidone, 1 mM dithiothreitol, 1 mM phenylmethylsulfonyl, pH 7.8). Extractions were centrifuged at 15,000 g for 30 min at 4°C and supernatant collected for subsequent enzyme assay and the quantification of malondialdehyde. Superoxide dismutase (EC 1.15.1.1) activity was determined by recording the rate of p-nitro blue tetrazolium chloride reduction at 560 nm [25] using a spectrophotometer (Ultrospec 2100, Biochrom LTD, UK). The activity of peroxidase (EC 1.11.1.7) and catalase (EC 1.11.1.6) was determined by measuring the decline in H_2_O_2_ expressed as changes in absorbance at 470 and 240 nm, respectively, using the spectrophotometer.

The level of membrane lipid peroxidation was determined weekly during salinity treatment by measuring the content of malondialdehyde (MDA) using the method of Dhindsa et al. [26] with modifications. A 0.8-ml aliquot of supernatant from the extract collected for antioxidant enzyme measurements was mixed with 1.6 ml 20% trichloroacetic acid containing 0.5% thiobarbituric acid. The mixture was heated at 95°C for 30 min, quickly cooled on ice, and then centrifuged at 10,000 g for 10 min. The absorbance of the supernatant was read at 532 and 600 nm using the spectrophotometer. The concentration of MDA was calculated using an extinction coefficient of 155 mM^−1^·m^−1^ [27].

### Content of sodium and potassium ion

The content of sodium (Na^+^) and potassium ions (K^+^) in leaves were determined using the method of Wang and Zhao [28] with modifications. Fresh leaf tissues were washed with distilled water immediately after harvesting, dried at 80°C for 72 h in an oven, and subsequently ground into fine powders with a mortar and pestle. About 0.2 g powder were added in 5 ml HNO_3_/HClO_4_ (v∶v = 4∶1) and then incubated at 80°C for 30 min, and then at 180°C until white smoke disappeared. After filtering the extracts, the content of both Na^+^ and K^+^ were assayed by flame emission (ICP-OES, Perkin Elmer Optima 2100DV, Perkin Elmer, USA).

### Metabolite analysis

Metabolites were extracted from leaf tissues collected at 28 d of salinity following the method used by Du et al. [29]. Leaf tissue samples were frozen in liquid nitrogen and stored at −80°C for metabolic profiling. The extraction protocol was modified from Roessner et al. [30] and Rizhsky et al. [31]. For each sample, frozen leaves were ground to a fine powder with liquid nitrogen, 25 mg leaf tissue powders were transferred into a 10 ml microcentrifuge tubes, and were extracted in 1.4 ml of 80% (v/v) aqueous methanol for 2 h at 200 rpm at ambient temperature. Ribitol solution of 10 µl (2 mg ml^−1^ water) was added as an internal standard prior to incubation. Then, extraction was done in a water bath at 70° for 15 min. Tubes were centrifuged for 30 min at 12,000 rpm, supernatant was decanted to new culture tubes, and 1.4 ml of water and 0.75 ml of chloroform were added. The mixture was vortexed thoroughly and centrifuged for 5 min at 5,000 rpm. 2 ml polar phase (methanol/water) was decanted into 1.5 ml HPLC vials respectively and dried in a Centrivap benchtop centrifugal concentrator. The dried polar phase was methoximated with 80 µl of 20 mg ml-1 methoxyamine hydrochloride at 30°C for 90 min and was trimethylsilylated with 80 µl N-Methyl-N-(trimethylsilyl)trifluoroacetamide (with 1% trimethylchlorosilane) for 60 min at 70°C.

Metabolites were separated and identified using gas chromatography and mass spectrometry, (GC-MS) analysis respectively, following the procedure modified from Qiu et al. [32]. The derivatized extracts were analyzed with a PerkinElmer gas chromatograph coupled with a TurboMass-Autosystem XL mass spectrometer (PerkinElmer Inc., USA). A 1 µl aliquot of the extracts was injected into a DB-5MS capillary column (30 m×0.25 mm×0.25 µm, Agilent J & W Scientific, Folsom, CA). The inlet temperature was set at 260°C. After a 6.5 min solvent delay, initial GC oven temperature was set at 60°C; 1 min after injection, the GC oven temperature was raised to 280°C for 5 min, and finally held at 280°C for 15 min. The injection temperature was set to 280°C and the ion source temperature was adjusted to 200°C. Helium was used as the carrier gas with a constant flow rate set at 1 ml min^−1^. The measurements were made with electron impact ionization (70 eV) in the full scan mode (m/z 30–550). The metabolites detected were identified by Turbomass 4.1.1 software (PerkinElmer Inc., USA) coupled with commercially available compound libraries: NIST 2005, Wiley 7.0. For GC/MS results, compounds were identified based on retention time and comparison with reference spectra in mass spectral libraries. Peaks areas of compounds were integrated with the Genesis algorithm. The relative quantity od metabolites were determined by comparing to the Ribitol internal standard.

## Results

The analysis of variant for the effects of ALA, salinity and interaction between ALA and salinity demonstrated significant effects of individual treatment on all parameters (Table 1). There are also significant interactive effects of ALA and salinity for majority of parameters measured in this study. The main effects and interactive effects of ALA and salinity for different parameters are presented below.

**Table 1:** Mean square values from analyses of variance of data for different parameters of ‘L93’creeping bentgrass at 28 d of experiment.

	TQ	SGR	RWC	Chl	EL	MDA
ALA	4.410*	0.221*	100.688*	0.321*	184.423*	32.174*
Salinity	31.923*	0.250*	2526.65*	0.275*	1700.155*	85.372*
ALA×Salinity	3.610*	0.087*	119.113*	0.0598*	257.672*	35.844*
Error	0.011	0.002	6.307	0.004	4.793	0.478

	Pn	gs	Tr	SOD	POD	CAT
ALA	7.185*	0.001ns	4.594*	0.007*	453.690*	3.950*
Salinity	8.002*	0.848*	546.070*	0.006*	540.563*	0.753*
ALA×Salinity	4.908*	0.008*	2.917*	0.0001*	16.000*	0.183ns
Error	0.207	0.001	0.541	4.583E-05	1.048	0.087

	Na^+^	K^+^	Na^+^/K^+^	Ket acid	Suc acid	Malic acid
ALA	112.486*	0.132ns	0.120*	0.0001*	0.006*	0.238*
Salinity	1428.774*	444.570*	1.673*	0.0001*	0.083*	0.019*
ALA×Salinity	81.224*	0.0001ns	0.094*	0.0001*	0.031*	0.046*
Error	0.725	4.58	0.0002	1.67E-07	0.0001	0.0007

	L-Alanine	Oxop	L-Asp	GABA	Sucrose	Glucose
ALA	1.225*	0.049*	0.005*	8.851*	26.176*	0.867*
Salinity	0.549*	0.662*	0.001*	5.297*	101.854*	8.861*
ALA×Salinity	0.275*	0.029*	0.001*	4.352*	53.051*	5.055*
Error	0.021	0.0003	7.65E-06	0.017	0.399	0.029

	Fructose	Lyxose	Allose	Galactose	Xylose	Maltose
ALA	0.731*	0.008*	0.0001*	0.0001*	0.018*	0.005*
Salinity	5.385*	0.012*	0.001*	0.002*	0.074*	0.001*
ALA×Salinity	4.502*	0.013*	0.0001ns	0.0001ns	0.005*	0.008*
Error	0.034	0.0002	2.65E-06	1.574E-05	3.71E-05	3.24E-05

* = significant at 0.05 level, ns = non-significant.

TQ = Turf quality, SGR = Shoot growth rate, RWC = Relative water content, Chl = Total chlorophyll content, EL = Electrolyte leakage, MDA = Malondialdehyde content, Pn = Net photosythesis rate, gs = Stomatal conductance, Tr = Transpiration rate, Ket = Ketoglutaric, Suc = Succinic, Oxop = Oxoproline, Asp = Aspartic acid.

### Effects of ALA in alleviating adverse salinity stress on growth, water status and photosynthesis

**Figure 1:**
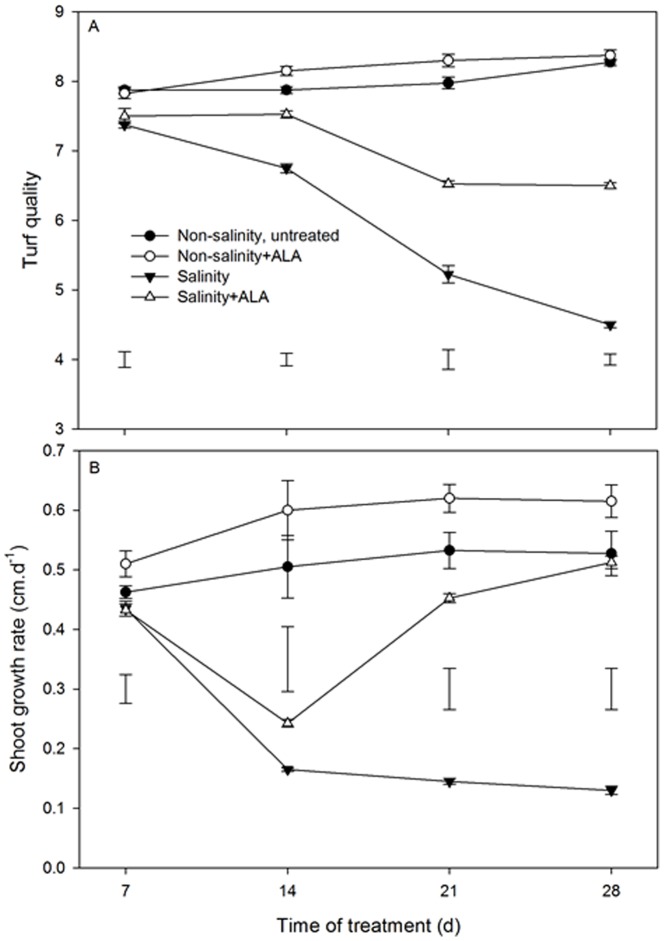
Turf quality (TQ) (A) and shoot growth rate (B) of ‘L93’creeping bentgrass for plants with ALA application (Nonsalinity+ALA) or without ALA application (Nonsalinity, untreated) under non-salinity conditions and salinity-stressed plants without ALA application (salinity) or with ALA application (salinity+ALA). The bars on the lines are standard error of the mean for four replicates. The vertical bars at the bottom of the figure represent least significant difference (LSD) values for comparison between treatments at a given day of treatment (*P*≤0.05).

Turf quality was maintained at approximately 8.0 in the plants under non-salinity treatment with or without ALA treatment (Fig. 1A). TQ began to decline significantly below the non-salinity, untreated level at 7 d in plants exposed to salinity alone without application of ALA or with ALA (salinity+ALA) (Fig. 1), but the decline was less pronounced for salinity+ALA plants than salinity alone treatment after 14 d. In addition, TQ continued to decline from 21 to 28 d for salinity treatment alone while that did not occur in salinity+ALA treatment.

Salinity for 28 d caused significant decline in SGR, but the decline was to a lesser extent for salinity+ALA plants than salinity alone (Fig. 1B). At 28 d of salinity stress, salinity+ALA plants maintained significantly greater SGR than plants exposed to salinity alone. Under non-salinity conditions, the application of ALA had no significant effects on SGR (Fig. 1B).

**Figure 2:**
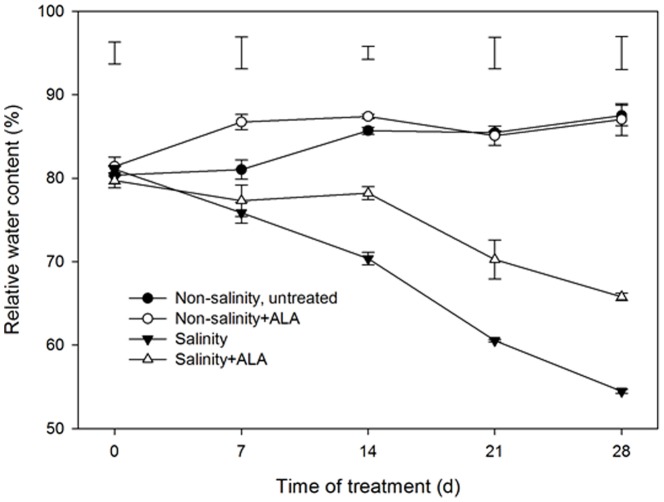
Leaf relative water content (RWC) of ‘L93’creeping bentgrass for plants with ALA application (Nonsalinity+ALA) or without ALA application (Nonsalinity, untreated) under non-salinity conditions and salinity-stressed plants without ALA application (salinity) or with ALA application (salinity+ALA). The bars on the lines are standard error of the mean for four replicates. The vertical bars at the top of the figure represent least significant difference (LSD) values for comparison between treatments at a given day of treatment (*P*≤0.05).

**Figure 3:**
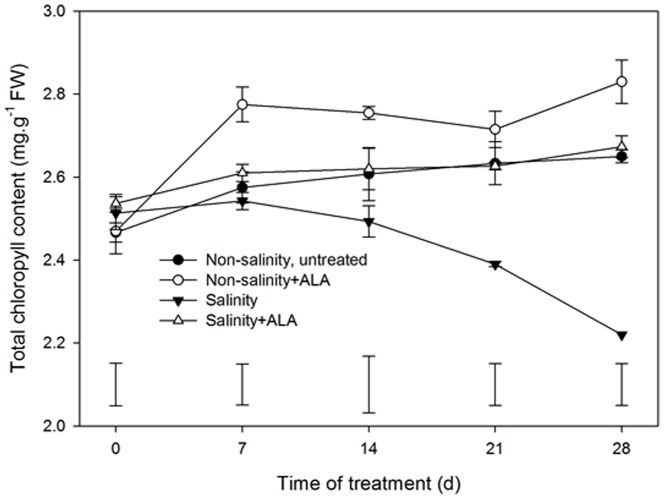
Leaf chlorophyll content of ‘L93’creeping bentgrass for plants with ALA application (Nonsalinity+ALA) or without ALA application (Nonsalinity, untreated) under non-salinity conditions and salinity-stressed plants without ALA application (salinity) or with ALA application (salinity+ALA). The bars on the lines are standard error of the mean for four replicates. The vertical bars at the bottom of the figure represent least significant difference (LSD) values for comparison between treatments at a given day of treatment (P≤0.05).

Leaf RWC was maintained at approximately 85% for nonsalinity, untreated and non-salinity+ALA treatments during the experiment (Fig. 2). A significant decline in RWC was observed, beginning at 7 d in either salinity+ALA or salinity alone treatment. After 14 d of treatment, salinity+ALA plants maintained significantly higher RWC than salinity treatment (Fig. 3).

A significant decline in Chl content was observed in salinity treatment (Fig. 3). Chl content was significantly greater in non-salinity+ALA and salinity+ALA plants than non-salinity, untreated and salinity alone, respectively. The positive effects of ALA were more pronounced under salinity than under non-salinity conditions.

**Figure 4:**
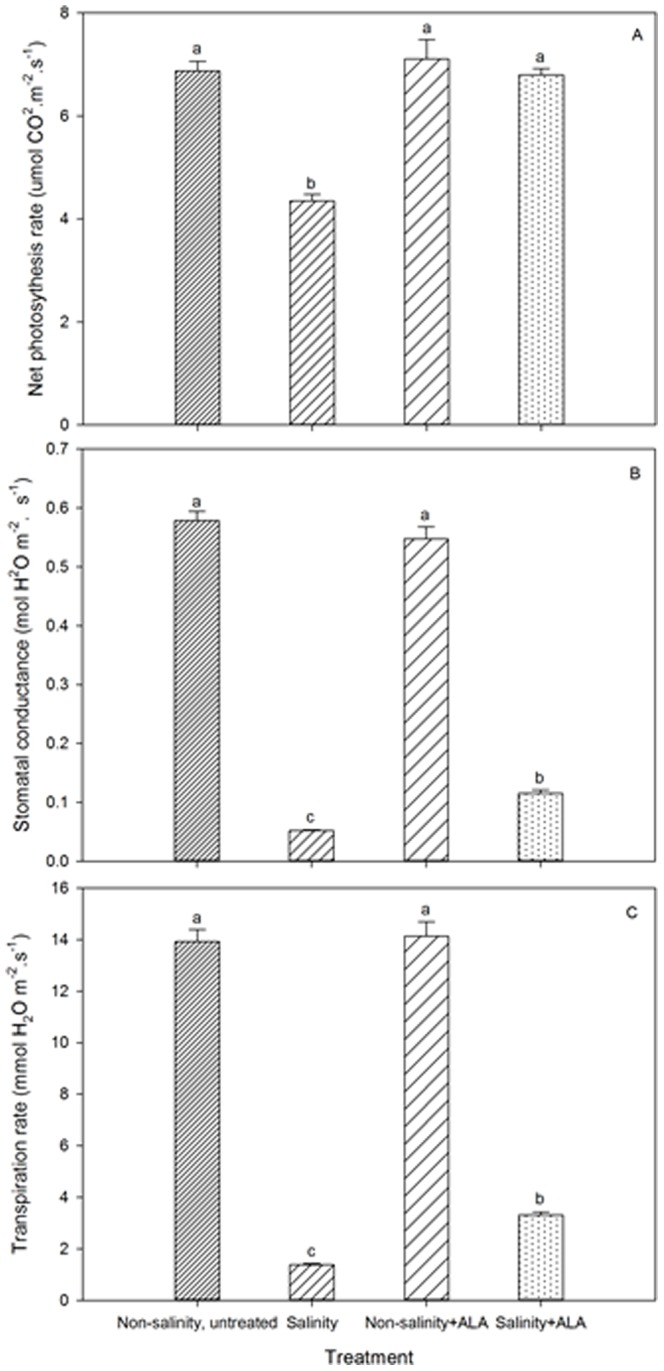
Leaf net photosynthetic rate (Pn), stomatal conductance (gs) and transpiration rate (Tr) of ‘L93’creeping bentgrass for plants with ALA application (Nonsalinity+ALA) or without ALA application (Nonsalinity, untreated) under non-salinity control conditions and salinity-stressed plants without ALA application (salinity) or with ALA application (salinity+ALA) at 28 d of salinity stress. Columns marked with different letters indicate significant differences based on least significant difference (LSD) test (P≤0.05).

Salinity treatment caused a significant decline in Pn, Tr, or g_s_ (Fig. 4). The application of ALA significantly increased Pn, Tr, or g_s_ of plants at 28 d of treatment (salinity+ALA vs. salinity in Fig. 4). Under non-salinity conditions, no significant differences in Pn, Tr, or g_s_ were detected between the untreated and ALA-treated plants at 28 d.

### Effects of ALA on salinity-induced cellular membrane damages and antioxidant enzyme activities

**Figure 5:**
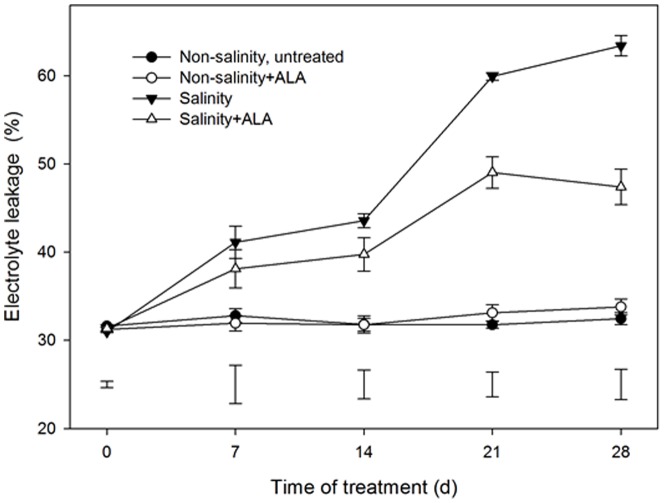
Leaf electrolyte leakage (EL) of ‘L93’creeping bentgrass for plants with ALA application (Nonsalinity+ALA) or without ALA application (Nonsalinity, untreated) under non-salinity conditions and salinity-stressed plants without ALA application (salinity) or with ALA application (salinity+ALA). The bars on the lines are standard error of the mean for four replicates. The vertical bars at the bottom of the figure represent least significant difference (LSD) values for comparison between treatments at a given day of treatment (P≤0.05).

Leaf EL increased in both salinity alone and salinity+ALA treatments, beginning at 7 d (Fig. 5). The application of ALA (salinity+ALA) significantly reduced EL of plants, beginning at 14 d of salinity stress and EL remained lower in salinity+ALA treatment than that in salinity alone at 21 and 28 d. No significant differences in EL between the untreated and ALA-treated plants under non-salinity conditions (Fig. 5).

**Figure 6:**
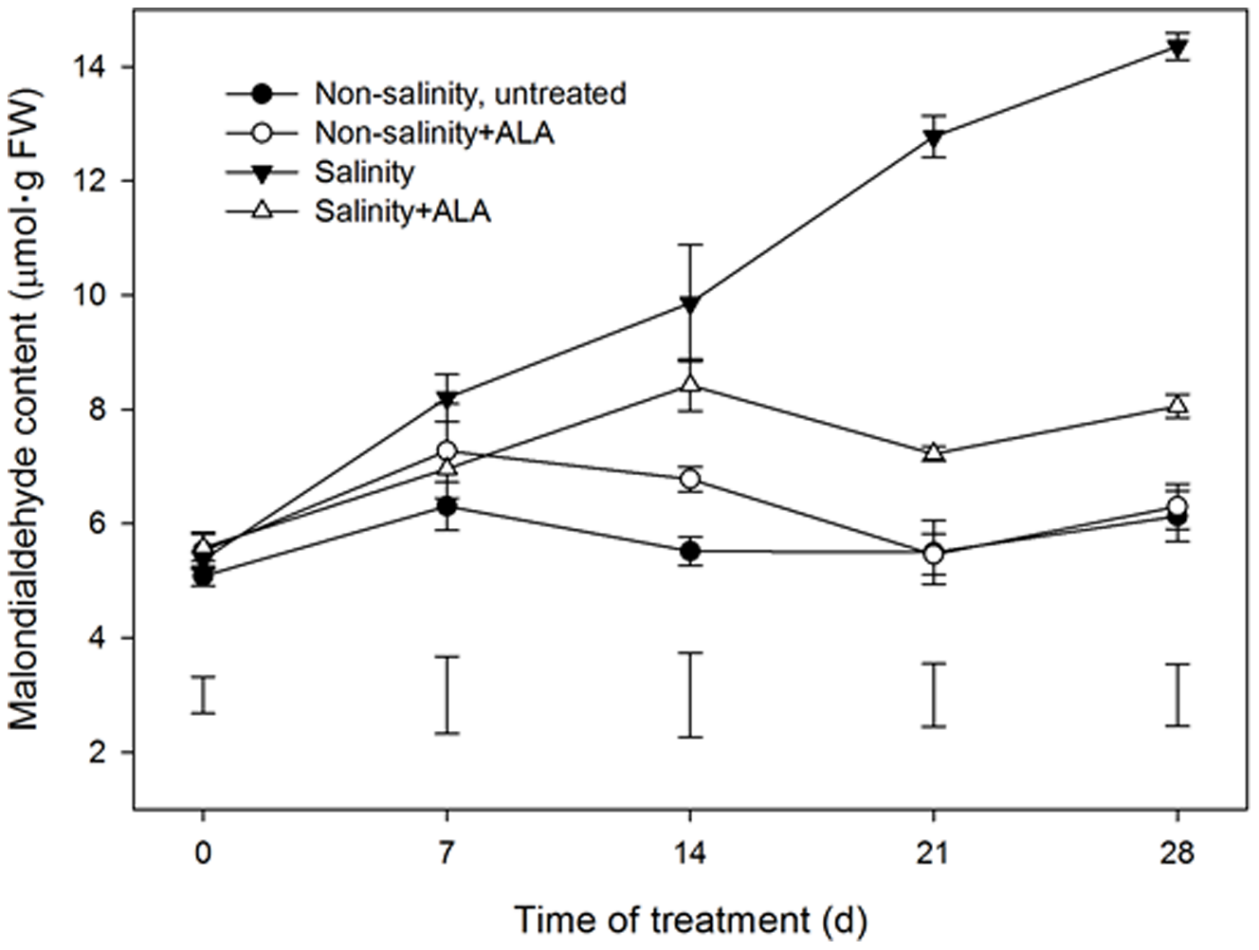
Leaf malondialdehyde (MDA) content of ‘L93’creeping bentgrass for plants with ALA application (Nonsalinity+ALA) or without ALA application (Nonsalinity, untreated) under non-salinity conditions and salinity-stressed plants without ALA application (salinity) or with ALA application (salinity+ALA). The bars on the lines are standard error of the mean for four replicates. The vertical bars at the bottom of the figure represent least significant difference (LSD) values for comparison between treatments at a given day of treatment (P≤0.05).

A significant increase in leaf MDA content was detected, beginning at 7 d in the salinity treatment and salinity+ALA plants, compared to the non-salinity, untreated plants (Fig. 6). The application of ALA resulted in a significantly lower MDA content during 28-d salinity stress, but had no significant effects under non-salinity conditions.

**Figure 7:**
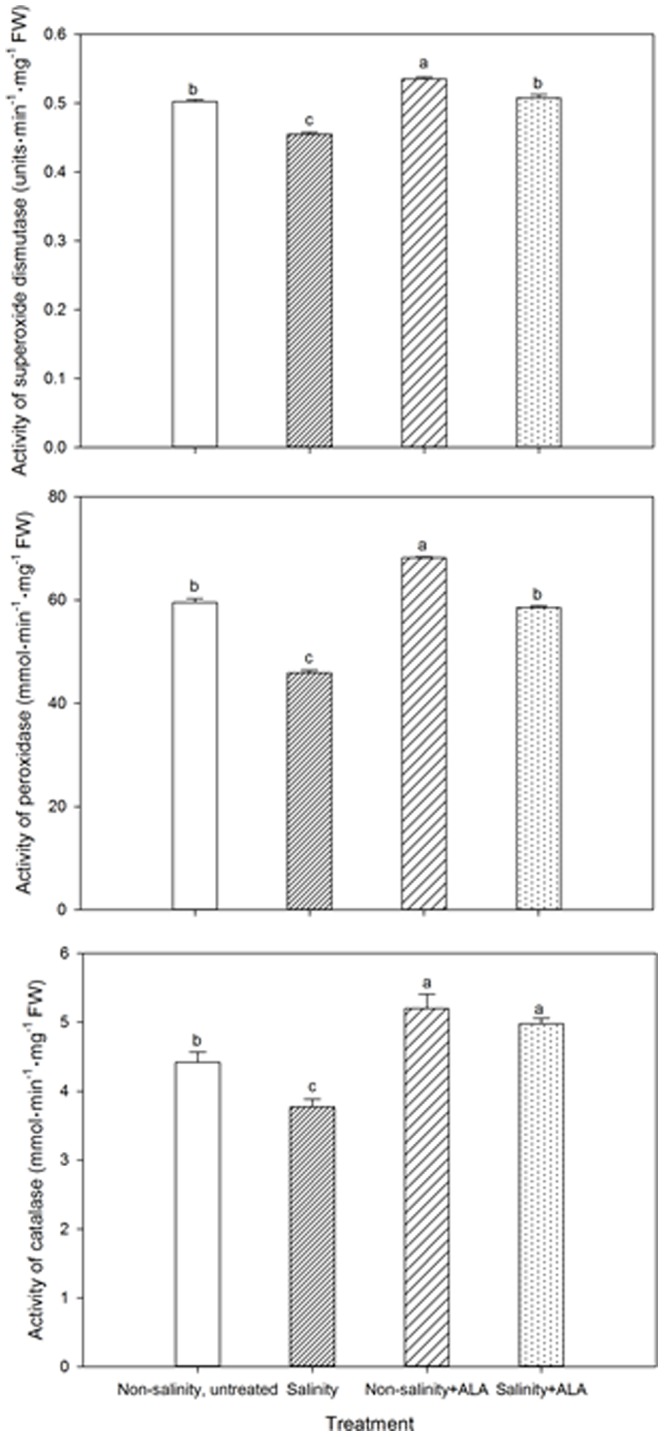
Activity of superoxide dismutase (SOD), peroxidase (POD) and catalase (CAT) of ‘L93’creeping bentgrass for plants with ALA application (Nonsalinity+ALA) or without ALA application (Nonsalinity, untreated) under non-salinity conditions and salinity-stressed plants without ALA application (salinity) or with ALA application (salinity+ALA) at 28 d of salinity. Columns marked with different letters indicate significant differences based on least significant difference (LSD) test (*P*≤0.05).

The activities of SOD, POD, and CAT of plants exposed to salinity alone decreased to significantly below the the non-salinity level by 28 d, while the enzyme activities in salinity+ALA plants had no difference from those in the non-salinity plants (Fig. 7). The activities of all three enzymes were significantly greater in ALA-treated plants compared to non-salinity, untreated plants and in salinity+ALA plants than salinity alone plants, and the enhanced activities were more pronounced under salinity than non-salinity conditions.

### Effects of ALA on Na^+^ and K^+^ accumulation in response to salinity stress

**Figure 8:**
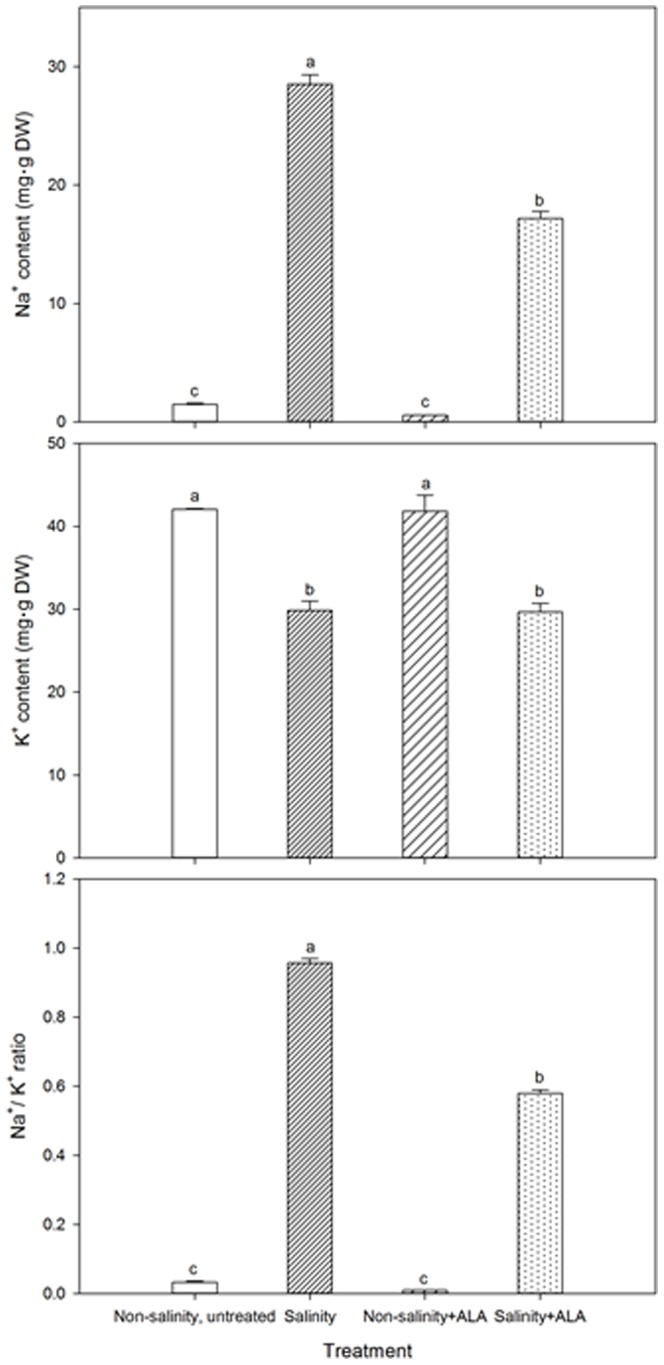
Leaf Na^+^ and K^+^ content and Na^+^/K^+^ ratio of ‘L93’creeping bentgrass for plants with ALA application (Nonsalinity+ALA) or without ALA application (Nonsalinity, untreated) under non-salinity conditions and salinity-stressed plants without ALA application (salinity) or with ALA application (salinity+ALA). Columns marked with different letters indicate significant differences based on least significant difference (LSD) test (*P*≤0.05).

The Na^+^ content and Na^+^/K^+^ ratio in leaves increased to significantly greater levels than the non-salinity treatment in both salinity alone and salinity+ALA treatments at 28 d (Fig. 8). The application of ALA (salinity+ALA) reduced Na^+^ content and Na^+^/K^+^ ratio at 28 d, compared to plants under salinity treatment without ALA, but had no effects on these two parameters under non-salinity stress. The K^+^ content decreased in both salinity and salinity+ALA treatments. The application of ALA did not cause significant changes in K^+^ content under salinity or non-salinity control conditions.

### Effects of ALA application metabolic responses to salinity stress

The GC-MS analysis detected 54 metabolites that were responsive to ALA or salinity treatments (Table 2), including 23 organic acids, 10 amino acids, and 21 sugars. Different metabolites exhibited differential responses to salinity and ALA treatments. Results of 15 metabolites that exhibited both significant decline with salinity and increases with ALA application, which are involved in important biological functions (energy, amino acid metabolism and osmotic adjustment) for plant tolerance to salinity stress, are described below. The metabolites that did not respond to the application of ALA are not presented here.

**Table 2:** List of identified metabolites in leaves of creeping bentgrass ‘L93’ in the control, ALA, salinity, or salinity+ALA treatments at 28 d of experiment.

	No.	Compound	Mass	Retention time (min)
Organic acids	1	2-Keto-l-gluconic acid	205	26.7991
	2	Aconitic acid	147	26.5608
	3	α-Ketoglutaric acid	55	28.4668
	4	Aminoadipic acid	217	36.9181
	5	Citric acid	273	27.9777
	6	Galacturonic acid	204	46.4101
	7	Gluconic acid	205	31.8272
	8	Glucopyranuronic acid	217	38.2723
	9	Glyceric Acid	189	16.4167
	10	Glycolic acid	147	9.44504
	11	Malic acid	204	35.8773
	12	Malonic acid	174	22.5985
	13	Methylglutaconic acid	147	24.6047
	14	Methylmaleic acid	67	18.2976
	15	Oxalic acid	147	11.1754
	16	Phosphonic acid	449	31.915
	17	Pyruvic acid	73	8.73031
	18	Shikimic acid	204	27.8649
	19	Succinic acid	147	15.9904
	20	Tartronic acid	217	32.0278
	21	Thiobarbituric acid	204	28.7928
	22	Trihydroxybutyric acid	205	22.2474
	23	Xylonic acid	117	24.2662
Amino acids	24	Aspartic acid	218	21.2819
	25	5-Oxoproline	156	21.1941
	26	Alanine	116	10.1598
	27	GABA	174	21.47
	28	Glutamic acid	246	23.6518
	29	Glutamine	73	27.012
	30	Glycine	147	15.7146
	31	Serine	204	17.2192
	32	Threonine	218	17.8838
	33	Valine	218	13.2444
sugars	34	Aldopentoses	205	22.7364
	35	Allose	205	33.6078
	36	Arabinofuranose	217	26.9119
	37	Arabinose	103	25.1188
	38	Cellobiose	145	35.0748
	39	D-Glycero-D-gulo-Heptose	191	37.9588
	40	Fructose	217	29.1062
	41	Fucose	117	25.7834
	42	Galactose	205	34.1219
	43	Glucose	205	29.6078
	44	Lyxose	217	24.9433
	45	Maltose	204	43.7644
	46	Mannobiose	204	40.9431
	47	Mannose	204	39.1876
	48	Melibiose	204	49.0809
	49	Sucrose	217	42.3725
	50	Talopyranose	191	38.8992
	51	Talose	204	31.2253
	52	Trehalose	191	43.8522
	53	Xylopyranose	205	28.6047
	54	Xylose	204	32.1532

**Figure 9:**
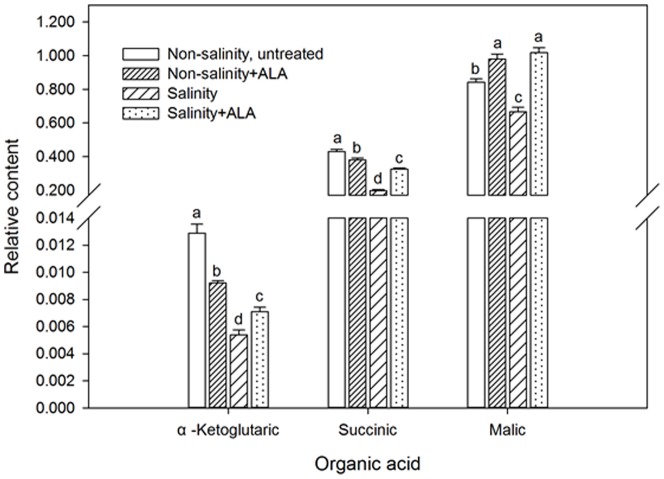
Organic acid content of ‘L93’creeping bentgrass for plants with ALA application (Nonsalinity+ALA) or without ALA application (Nonsalinity, untreated) under non-salinity conditions and salinity-stressed plants without ALA application (salinity) or with ALA application (salinity+ALA) at 28 d of salinity. Columns marked with different letters indicate significant differences based on least significant difference (LSD) test (*P*≤0.05).

Salinity stress caused significant decline in the content of several organic acids involved in respiration. Those organic acids exhibited decline (by percentage from the control) with salinity included α-ketoglutaric acid (by 58.1%), succinic acid (by 54.0%), and malic acid (by 20.9%). The salinity+ ALA plants had significantly higher content of those organic acids compared to the plants exposed to salinity alone (Fig. 9).

**Figure 10:**
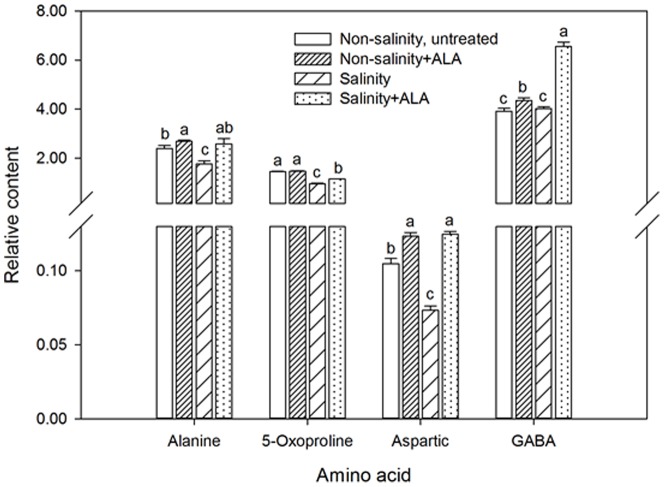
Amino acid content accumulation ‘L93’creeping bentgrass for plants with ALA application (Nonsalinity+ALA) or without ALA application (Nonsalinity, untreated) under non-salinity conditions and salinity-stressed plants without ALA application (salinity) or with ALA application (salinity+ALA) at 28 d of salinity. Columns marked with different letters indicate significant differences based on least significant difference (LSD) test (*P*≤0.05).

For amino acids, the content of alanine, 5-oxaproline, aspartic acid was decreased by salinity stress (Fig. 10). The content of those three amino acids were significantly higher in salinity+ALA plants than those in salinity alone treatment. For γ -aminobutyric acid (GABA), the abundance was significantly increased by ALA under salinity stress while salinity stress did not affect its content (Fig. 10). Many other amino acids were detected in this experiment (Table 2), but the content was not affected significantly by ALA (data not shown).

**Figure 11:**
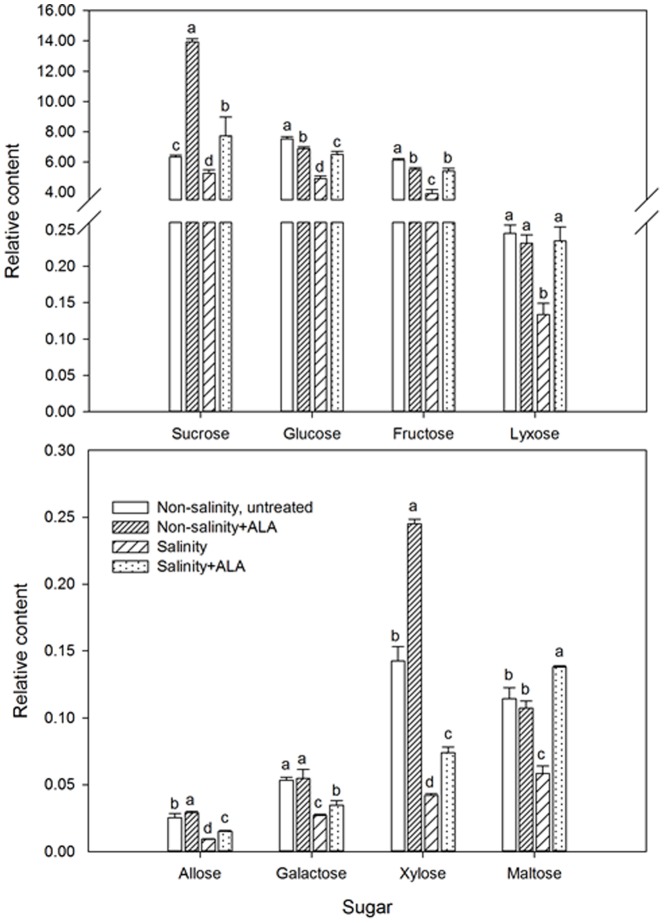
Sugar content ‘L93’creeping bentgrass for plants with ALA application (Nonsalinity+ALA) or without ALA application (Nonsalinity, untreated) under non-salinity conditions and salinity-stressed plants without ALA application (salinity) or with ALA application (salinity+ALA) at 28 d of salinity. Columns marked with different letters indicate significant differences based on least significant difference (LSD) test (P≤0.05).

Salinity stress caused a decline in the abundance of majority type of sugars compared to the non-salinity control, including sucrose (by 17.1%), glucose (by 34.7%), fructose (by 36.2%), lyxose (by 45.5%), allose (by 63.4%), galactose (by 49.3%), xylose (by 70.4%) and maltose (by 48.9%) (Fig. 11). The application of ALA enhanced the accumulation of those sugars under salinity stress (Fig. 11).

## Discussion

The growth parameters (TQ and SGR) and physiological analyses (RWC, Chl, Pn, g_s_ and Tr) demonstrated that foliar application of ALA at 0.5 mg L^−1^ effectively mitigated growth and physiological damages due to salinity. To our knowledge, this is the first report of positive effects of ALA on perennial grass species responses to salinity. The greater RWC suggested that ALA facilitated the maintenance of cellular hydration despite osmotic stress under salinity. Enhanced chlorophyll content could contribute to the increases in Pn by ALA that may promote the light harvesting capacity [7]. Plants treated with ALA were able to maintain greater g_s_ and Tr under salinity, which could be associated with greater RWC and also indicated that ALA may reduce stomatal limitation to gas exchanges, thereby also contributing to the increases in Pn. Enhanced Pn by ALA has also been attributed to the reduction in stomatal limitation in date palm seedlings (*Phoenix dactylifera*) exposed to salinity stress [7]. Salinity-induced decline in Pn can also be due to metabolic or non-stomatal limitations, such as decline in chlorophyll content controlling light harvesting capacity and inhibition of enzyme activities involved in carboxylation and carbon assimilation [3]. The maintenance of regular shaped chloroplasts with more intact thylakoids with ALA has been related to enhanced photosynthesis in *Brassica napus* [9]. In this study, the increased chlorophyll content by ALA in creeping bentgrass exposed to salinity stress could enhance light-harvesting capacity, leading to greater Pn. However, whether the enhanced Pn by ALA in creeping bentgrass exposed to salinity stress could be associated with metabolic factors involved in carboxylation and carbon assimilation is unclear, which deserves further investigation. The enhancement in salinity tolerance by ALA in creeping bentgrass, as manifested by growth and physiological stimulation, could be associated with the reduction in Na^+^ accumulation, cellular membrane protection, antioxidant enzyme activities, and accumulation of metabolites for osmotic adjustment and energy metabolism, as discussed in detail below.

Salinity stress is characterized by Na^+^ toxicity and ion imbalance, which is caused by the replacement of K^+^ by Na^+^ in plants sensitive to salinity stress [33]. The maintenance of low Na^+^/K^+^ ratio in plants is one of the major characteristics of salinity tolerance [34]. A Na^+^/K^+^ ratio equal or lower than 0.6 is considered optimal for maintaining normal metabolic processes in non-halophyte plants [35]. In this study, salinity stress increased Na^+^ content and decreased K^+^ content, leading to increases in Na^+^/K^+^ ratio approaching 1.0 in creeping bentgrass. However, foliar application of ALA significantly reduced Na^+^ content in leaves, although it had no effects on K^+^, resulting in lower Na^+^/K^+^ ratio (less than 0.6). Watanabe et al. [6] did not find significant effects of ALA on Na^+^ transport from roots to shoots, but reported the reduction in Na^+^ content in roots of cotton plants treated with ALA, suggesting that ALA application may limit Na^+^ uptake, although the underlying mechanisms are unclear. Reduction in Na^+^ content and the ratio of Na^+^/K^+^ has been reported in *Brassica napus* [9]. Our results suggested that ALA did not affect K^+^ accumulation, but could suppress root uptake of Na^+^ and/or transport of Na^+^ from roots to leaves, resulting in lower Na^+^/K^+^ ratio and thereby mitigating Na^+^ toxicity in leaves in creeping bentgrass.

The accumulation of Na^+^ also caused damages in cellular membranes, as manifested by increased EL and MDA under salinity stress in creeping bentgrass in this study, and in other plant species reported in previous studies, such as *Brassica napus* [15]. Positive effects of ALA in stress responses could be associated with the protective roles in cellular membranes, as shown by the decreased EL and MDA content in ALA-treated plants compared to untreated plants under salinity stress in this study. Reduction in lipid peroxidation by ALA application has also been reported in other plant species, such as watermelon (*Citrullus lanatus*) [36], rice (*Oryza sativa*) [37], and *Brassica napus* [15]. Maintenance of cell membrane stability could be attributed in part to the effects of ALA application on the antioxidant enzyme responses to prevent lipid peroxidation. Plant antioxidant systems are comprised of a network of multiple enzymes that function to detoxify the various forms of reactive oxygen species that cause lipid peroxidation, including SOD catalyzing the conversion of highly reactive superoxide to hydrogen peroxide [24], POD and CAT involved in converting hydrogen peroxide into O_2_ and H_2_O within the glutathione-ascorbate cycle [38]. Foliar application of ALA enhanced the activities of SOD, POD, and CAT for creeping bentgrass under salinity stress in this study, which is consistent with results reported in other plant species [36], [37], [39], [40]. Zhen et al. [39] reported that ALA decreased the H_2_O_2_ content and increased the gene expression level and enzymatic activity of CAT in roots and leaves of cucumber (*Cucumis sativus*) under salinity stress. Enhanced activities of antioxidant enzymes by ALA treatment were also reported in plants growing under other environmental conditions, such as *Brassica napus* under herbicide toxicity stress [41] and watermelon seedlings exposed to low light conditions [42]. The results in creeping bentgrass along with studies in other plant species strongly support the notion that foliar ALA was effective in suppressing oxidative damage induced by salinity stress by affecting antioxidant enzyme activity. The mechanisms of how ALA may activate antioxidant defense systems are not yet clear. Naeem et al. [15] postulated that the enhanced activities of antioxidant enzymes is possibly the result of the stimulation of heme-based biomolecules since ALA is the essential precursor of heme biosynthesis and APX and CAT are composed of hemes.

Respiratory metabolism is essential for plant growth and stress tolerance. However, salinity stress in creeping bentgrass inhibited the production of several major organic acids involved in the tricarboxylic acid cycle of respiration, including α-ketoglutaric acid, succinic acid, and malic acid in this study. Salinity-induced reduction in the accumulation of those three organic acids have also been reported in other plant species, such as *Arabidopsis*, rice, and barley (*Hordeum spp.*) in previous studies [43], [44], [45]. The decline in organic acid accumulation may be associated with the suppression of respiratory energy production under salinity stress. However, salinity-induced decline in α-ketoglutaric acid, succinic acid, and malic acid was mitigated by ALA application, which could be reflective of enhanced respiratory activity under salinity stress.

Salinity damages may involve changes in amino acid metabolism, as shown by the reduction in the content of amino acids, such as valine, glycine, proline, serine, threonine and aspartate in wild barley (*Hordeum spontaneum*) [45] or increased content of leucine and isoleucine in *Arabidopsis thaliana* and *Lotus japonicas* under salinity stress [43]. In this study, salinity caused significant reduction in the abundance level of alanine, 5-oxoproline, and aspartic acid, and did not have significant effects on other amino acids (data not shown). Alanine is involved in protein and sugar metabolism [46]. In both wild and cultivated barley, the salt-induced decline in alanine was attributed to the inhibition of pyruvate metabolism [45]. 5-oxoproline is a non-protein amino acid involved in protein storage and antioxidant systems, which has been associated with plant adaptation to abiotic stresses [47], [48], [49]. Aspartatic acid serves as a common precursor of some essential amino acids, such as lysine, threonine, methionine and isoleucine in plants [50]. While salinity inhibited the accumulation of alanine, 5-oxoproline, and aspartic acid, the application of ALA enhanced the accumulation of those amino acids under salinity stress. In addition, GABA content also increased in ALA-treated plants under non-salinity and salinity conditions, although salinity had no significant effects on its content. GABA functions in various pathways such as pH regulation, osmotic adjustment, defense system, carbon and nitrogen metabolism [51], [52]. The enhanced accumulation of alanine, 5-oxaproline, aspartic acid, and GABA due to ALA application under salinity stress in creeping bentgrass might be associated with some important metabolic pathways involved in osmotic adjustment, cellular membrane and protein integrity and stability [45], [53].

Salinity stress caused a significant decrease in the content of monosaccharides, including glucose, fructose, galactose, lyxose, allose and xylose and disaccharides sucrose, and maltose in creeping bentgrass in this study. Similar results have been reported in other plant species exposed to salinity stress, such as *Arabidopsis thaliana*, *Lotus japonicas* and *Oryza sativa* [43], *Hordeum spontaneum* and *Hordeum vulgare* [45]. Monosaccharides play important roles in energy supply for many metabolic processes and are involved in osmotic adjustment in plants, whereas disaccharides mainly serves as carbohydrates for transport and storage [54], [55], [56]. The effects of ALA on the accumulation of monosaccharides and disaccharides under stress conditions were not previously reported. In this study, foliar application of ALA enhanced the abundance of glucose, fructose, galactose, lyxose, allose, xylose, sucrose, and maltose under salinity stress, which could contribute to the improved salinity tolerance through promoting osmotic adjustment, energy reserves, and carbon metabolism.

In summary, this study demonstrated that exogenous application of ALA was effective for mitigating salinity damages in creeping bentgrass. The effects of ALA in creeping bentgrass were associated with the suppression of Na+ toxicity and enhanced water retention, photosynthetic activities, antioxidant metabolism, and the accumulation of organic acids, amino acids, and sugars involved in osmotic regulation and respiratory pathways, and protein metabolism, as well as stress protection from membrane damages. Further research at molecular levels may be necessary to identify molecular and metabolic pathways conferring ALA effects on stress mitigation in perennial grass and other plant species. In addition, field tests should be conducted to further examine the feasibility and effectiveness of ALA to promote plant tolerance to salinity under natural environmental conditions.
